# Supplementary material for: SARS-CoV-2 Is a Culprit for Some, but Not All Acute Ischemic Strokes: A Report from the Multinational COVID-19 Stroke Study Group
Source: J Clin Med. 2021 Mar 1;10(5):931. doi: 10.3390/jcm10050931 (PMC7957755; doi:10.3390/jcm10050931)
Supplement: Supplementary file 1 [file jcm-10-00931-s001.pdf]

Supplemental Material

Index

- Supplemental Document 1. Search Strategy in PubMed
- Supplemental Document 2. Detailed Description of the Clustering
- Supplemental Table 1. Dividing the SARS-CoV-2 infected acute ischemic stroke patients into 4 and 5 subgroups based on K-Mean clustering
- Supplemental Table 2. Dividing the SARS-CoV-2 infected acute ischemic stroke patients into 4 and 5 subgroups by Spectral clustering
- Supplemental Table 3. The characteristics of SARS-CoV-2 infected stroke patients in original dataset and literature review
- Supplemental 4. The characteristics of the patients from the literature review dataset divided based on clinical risk scoring models
- Supplemental 5. The characteristics of the patients from the literature review dataset divided based on unsupervised machine learning models
- Supplemental Figure 1. Contingency Matrices
- The COVID-19 Stroke Study Group

SARS-CoV-2 is a Culprit for Some, but not All Acute Ischemic Strokes

A Report from the Multinational COVID-19 Stroke Study Group

Authors

Shima Shahjouei, MD, MPH,[1] Michelle Anyaehie, MBS,[1] Eric Koza, MD Candidate,[2] Georgios Tsivgoulis, MD, PhD, MSc,[3] Soheil Naderi, MD,[4] Ashkan Mowla, MD,[1,5] Venkatesh Avula, MS,[1] Alireza Vafaei Sadr, PhD,[6] Durgesh Chaudhary, MBBS,[1] Ghasem Farahmand, MD,[7] Christoph Griessenauer, MD,[1,8] Mahmoud Reza Azarpazhooh, MD,[9] Debdipto Misra, MS,[10] Jiang Li, MD,[11] Vida Abedi, PhD,[11,12] Ramin Zand, MD, MPH[1];

the Multinational COVID-19 Stroke Study Group

[1]Neurology Department, Neuroscience Institute, Geisinger Health System, Danville, Pennsylvania, USA;

[2]Geisinger Commonwealth School of Medicine, Scranton, Pennsylvania, USA;

[3]Second Department of Neurology, National and Kapodistrian University of Athens, School of Medicine, "Attikon" University Hospital, Athens, Greece;

[4]Department of Neurosurgery, Tehran University of Medical Sciences, Tehran, Iran;

[5]Division of Stroke and Endovascular Neurosurgery, Department of Neurological Surgery, Keck School of Medicine, University of Southern California, California, USA;

[6]Department de Physique Theorique and Center for Astroparticle Physics, University Geneva, Switzerland;

[7]Iranian Center of Neurological Research, Neuroscience Institute, Tehran University of Medical Sciences, Tehran, Iran;

[8] Research Institute of Neurointervention, Paracelsus Medical University, Salzburg, Austria;

[9]Neurology Department, Western University, Canada;

[10]Steele Institute of Health & Innovation, Geisinger Health System, Pennsylvania, USA;

[11]Department of Molecular and Functional Genomics, Geisinger Health System, Danville, Pennsylvania, USA;

[12]Biocomplexity Institute, Virginia Tech, Blacksburg, Virginia, USA.

Corresponding Author:

Ramin Zand M.D., M.P.H.

Neuroscience Institute, Geisinger,

100 North Academy Ave. Danville, PA, 17822

phone number: 570-214-4101

Fax number: (570) 808-3208

E-mail address: rzand@geisinger.edu; ramin.zand@gmail.com

Laboratory: www.thedecodelab.com

Running Title: Stroke and COVID-19, Panel of Comorbidities

Funding and Grant Support: None

Word Count: 3385

Tables: 3

Figures: 1

Supplemental file: 1

Key Words: Cerebrovascular Disorders, Stroke, SARS-CoV-2, COVID-19, Cluster Analysis, Risk Factors, Comorbidity.

SUPPLEMENTAL DOCUMENT 1. SEARCH STRATEGY IN PUBMED

1. "Cerebrovascular Disorders"[MeSH Terms]
2. "Brain Ischemia"[MeSH Terms] OR "Hypoxia-Ischemia, Brain"[MeSH Terms] OR "Ischemic Attack, Transient"[MeSH Terms]
3. "Stroke"[MeSH Terms] OR "Stroke, Lacunar"[MeSH Terms] OR "Infarction, Posterior Cerebral Artery"[MeSH Terms] OR "Brain Stem Infarctions"[MeSH Terms] OR "Infarction, Middle Cerebral Artery"[MeSH Terms] OR "Infarction, Anterior Cerebral Artery"[MeSH Terms]
4. Stroke[Title/Abstract] OR cerebr vascul infarct[Title/Abstract] OR cerebrovasc infarct[Title/Abstract] OR cerebr vasc event[Title/Abstract] OR cerebrovasc event[Title/Abstract] OR CVA[Title/Abstract] OR transient ischemic attack[Title/Abstract]
5. “Intracranial Hemorrhages”[MeSH Terms] OR “Subarachnoid Hemorrhage”[MeSH Terms] OR “Putaminal Hemorrhage”[MeSH Terms] OR “Putaminal Hemorrhage”[MeSH Terms] OR “Basal Ganglia Hemorrhage”[MeSH Terms]
6. intra?cerebral hemorrhage[Title/Abstract] OR intra?parenchymal hemorrhage[Title/Abstract] OR “SAH” [Title/Abstract]
7. “venous sinus thrombosis, cranial”[MeSH Terms] OR “Cranial Sinuses”[MeSH Terms]
8. “cerebral venous thrombosis”[Title/Abstract] OR “cerebral sinus thrombosis” [Title/Abstract] OR “CVT” [Title/Abstract]
9. Neurologic[Title/Abstract]
10. #1 OR #2 OR #3 OR #4 OR #5 OR #6 OR #7 OR #8
11. COVID-19 [Supplementary Concept] OR “severe acute respiratory syndrome coronavirus 2 [Supplementary Concept]
12. SARS-CoV-2[Title/Abstract] OR COVID-19[Title/Abstract] OR coronavirus covid-19[Title/Abstract]
13. #10 OR #11

("Cerebrovascular Disorders"[MeSH Terms] OR "Brain Ischemia"[MeSH Terms] OR "Hypoxia-Ischemia, Brain"[MeSH Terms] OR "Ischemic Attack, Transient"[MeSH Terms] OR "Stroke"[MeSH Terms] OR "Stroke, Lacunar"[MeSH Terms] OR "Infarction, Posterior Cerebral Artery"[MeSH Terms] OR "Brain Stem Infarctions"[MeSH Terms] OR "Infarction, Middle Cerebral Artery"[MeSH Terms] OR "Infarction, Anterior Cerebral Artery"[MeSH Terms] OR Stroke[Title/Abstract] OR cerebr vascul infarct[Title/Abstract] OR cerebrovasc infarct[Title/Abstract] OR cerebr vasc event[Title/Abstract] OR cerebrovasc event[Title/Abstract] OR CVA[Title/Abstract] OR transient ischemic attack[Title/Abstract] OR "Intracranial Hemorrhages"[MeSH Terms] OR "Subarachnoid Hemorrhage"[MeSH Terms] OR "Putaminal Hemorrhage"[MeSH Terms] OR "Putaminal Hemorrhage"[MeSH Terms] OR "Basal Ganglia Hemorrhage"[MeSH Terms] OR intra?cerebral hemorrhage[Title/Abstract] OR intra?parenchymal hemorrhage[Title/Abstract] OR "SAH" [Title/Abstract] OR "venous sinus thrombosis, cranial"[MeSH Terms] OR "Cranial Sinuses"[MeSH Terms] OR "cerebral venous thrombosis"[Title/Abstract] OR "cerebral sinus thrombosis" [Title/Abstract] OR "CVT" [Title/Abstract] OR Neurologic[Title/Abstract]) AND (COVID-19 [Supplementary Concept] OR "severe acute respiratory syndrome coronavirus 2 [Supplementary Concept] OR SARS-CoV-2[Title/Abstract] OR COVID-19[Title/Abstract] OR coronavirus covid-19[Title/Abstract])

SUPPLEMENTAL DOCUMENT 2. DETAILED DESCRIPTION OF THE CLUSTERING

The patients were clustered based on the laboratory findings and comorbidities. We used clinicians' EXpert opinion (EX models), or unsupervised Machine Learning algorithms (ML models) for defining the clusters. The results of different models were compared through the contingency matrix (a.k.a contingency table).[3] The models for clustering the patients based on the comorbidities are as follows:

A. Clinical Risk Scoring Models Based on the EXpert opinion (EX models)

Patients were categorized based on the number of the existing comorbidities. We either considered All eleven comorbidities (EX-A models; including hypertension, diabetes mellitus, ischemic heart disease, atrial fibrillation, carotid stenosis, chronic kidney disease, cardiac ejection fraction less than 40%, active neoplasm, rheumatological diseases, history of transient ischemic attack or stroke, and current smoking), or Selected number of vascular comorbidities (EX-S models; including hypertension, diabetes mellitus, ischemic heart disease, atrial fibrillation, carotid stenosis, chronic kidney disease, previous transient ischemic attack or stroke, and current smoking). We further clustered the patients under each of these models to two ($EX-A_2$ and $EX-S_2$) or three ($EX-A_3$ and $EX-S_3$) subgroups.

1. $EX-A_2$: based on expert opinion and including all comorbidities, in two subgroups.
    a. Subgroup a: zero or one comorbidity,
    b. Subgroup b: more than one comorbidity.
2. $EX-S_2$: based on expert opinion and including selected comorbidities, in two subgroups.
    a. Subgroup a: zero or one comorbidity,
    b. Subgroup b: more than one comorbidity.
3. $EX-A_3$: based on expert opinion and including all comorbidities, in three subgroups.
    a. Subgroup a: zero comorbidity,
    b. Subgroup b: one or two comorbidities,
    c. Subgroup c: more than two comorbidities.
4. $EX-S_3$: based on expert opinion and including selected comorbidities, in three subgroups.
    a. Subgroup a: zero comorbidity,
    b. Subgroup b: one or two comorbidities,
    c. Subgroup c: more than two comorbidities.

B. Models Based on Machine Learning Algorithms (ML models)

We used unsupervised machine learning algorithms for clustering. Hierarchical (complete linkage method) and K-means (Hartigan-Wong algorithm) clustering (ML-K models) and Spectral clustering (ML-S models) were used. We grouped the patients into two ($ML-K_2$ and $ML-S_2$), three ($ML-K_3$ and $ML-S_3$), four ($ML-K_4$ and $ML-S_4$), and five ($ML-K_5$ and $ML-S_5$) clusters.

B1: Hierarchical and k-mean clustering

1. ML-K_2: Based on hierarchical and K-means clustering, dividing the patients into two subgroups.
2. ML-K_3: Based on hierarchical and K-means clustering, dividing the patients into three subgroups.
3. ML-K_4: Based on hierarchical and K-means clustering, dividing the patients into four subgroups.
4. ML-K_5: Based on hierarchical and K-means clustering, dividing the patients into five subgroups.

B2: Spectral clustering

1. ML-$S_{2:}$ Based on spectral clustering, dividing the patients into two subgroups.
2. ML-$S_{3:}$ Based on spectral clustering, dividing the patients into three subgroups.
3. ML-$S_{4:}$ Based on spectral clustering, dividing the patients into four subgroups.
4. ML-$S_{5:}$ Based on spectral clustering, dividing the patients into five subgroups.

SUPPLEMENTAL TABLE 1. DIVIDING THE SARS-COV-2 INFECTED ACUTE ISCHEMIC STROKE PATIENTS INTO 4 AND 5 SUBGROUPS BASED ON K-MEAN CLUSTERING

The table shows the structure of the models for grouping the patients into 4 and 5 subgroups using machine learning (ML) models (K-Mean) by considering the present comorbidities. ML-S_4 used spectral clustering to divide the patients into 4 groups while ML-S_5 separated the patients into 5 subgroups.

	Machine Learning Algorithm, K-Mean										
	ML-K_4 (K-Mean)					ML-K_5 (K-Mean)					
	a N= 74 (25.3%)	b N= 95 (32.5%)	c N= 62 (21.2%)	d N= 61 (20.9 %)	P-Value	a N= 85 (29.1%)	b N= 39 (18.8%)	c N= 55 (18.8%)	d N= 49 (16.8%)	e N=64 (21.9%)	P-Value
**Hypertension (%)**	100.0	0.0	87.1	83.6	<0.001	0.0	74.4	81.8	83.7	100.0	<0.001
**Diabetes Mellitus (%)**	0.0	0.0	58.1	100.0	<0.001	0.0	33.3	100.0	59.2	0.0	<0.001
**Ischemic Heart Disease (%)**	0.0	9.5	100.0	0.0	<0.001	5.9	43.6	0.0	100.0	0.0	<0.001
**Atrial Fibrillation (%)**	13.5	8.4	27.4	9.8	0.006	0.0	100.0	0.0	4.1	0.0	<0.001
**Carotid Stenosis (%)**	10.8	4.2	29.0	13.1	<0.001	4.7	5.1	12.7	34.7	12.5	<0.001
**Chronic Kidney Disease (%)**	20.3	12.6	6.5	16.4	0.12	12.9	7.7	16.4	8.2	21.9	0.178
**Cardiac Ejection Fraction<40% (%)**	12.2	1.1	14.5	8.2	0.01	1.2	20.5	7.3	12.2	7.8	0.006
**Active Neoplasm (%)**	5.4	1.1	21.0	4.9	<0.001	0.0	7.7	5.5	22.4	6.3	<0.001
**Rheumatological Disease (%)**	4.1	1.1	0.	1.6	0.29	0.0	5.1	0.0	0.0	4.7	0.05
**Previous Stroke/TIA (%)**	1.4	1.1	3.2	1.6	0.76	1.2	2.6	1.8	2.0	1.6	0.99
**Current Smoker (%)**	23.0	7.4	25.8	13.1	0.006	5.9	7.7	14.5	36.7	21.9	<0.001

SUPPLEMENTAL TABLE 2. DIVIDING THE SARS-COV-2 INFECTED ACUTE ISCHEMIC STROKE PATIENTS INTO 4 AND 5 SUBGROUPS BY SPECTRAL CLUSTERING

The table shows the structure of the models for grouping the patients into 4 and 5 subgroups using machine learning (ML) models (spectral clustering) by considering the present comorbidities. ML-S_4 used spectral clustering to divide the patients into 4 groups while ML-S_5 separated the patients into 5 subgroups.

Models	Machine Learning Algorithm, Spectral										
	ML-S_4 (Spectral)					ML-S_5 (Spectral)					
Comorbidities	a<br>N = 57<br>(19.3%)	b<br>N = 112<br>(37.8%)	c<br>N = 38<br>(12.8%)	d<br>N = 89<br>(30.1%)	P-value	a<br>N = 20<br>(6.8%)	b<br>N = 89<br>(30.1%)	c<br>N = 112<br>(37.8%)	d<br>N = 19<br>(6.4%)	e<br>N = 56<br>(18.9%)	P-value
Hypertension (%)	100.0	0.0	89.5	100.0	<0.001	95.0	100.0	0.0	84.2	100.0	<0.001
Diabetes Mellitus (%)	100.0	11.6	73.7	0.0	<0.001	85.0	0.0	13.4	52.6	100.0	<0.001
Ischemic Heart Disease (%)	10.5	11.6	100.0	16.9	<0.001	95.0	16.9	11.6	100.0	10.7	<0.001
Atrial Fibrillation (%)	10.5	8.0	44.7	11.2	<0.001	10.0	11.2	6.3	89.5	10.7	<0.001
Carotid Stenosis (%)	14.0	3.6	44.7	10.1	<0.001	85.0	10.1	3.6	5.3	12.5	<0.001
Chronic Kidney Disease (%)	14.0	12.5	7.9	18.0	0.46	15.0	18.0	12.5	0.0	14.3	0.34
Cardiac Ejection Fraction<40% (%)	7.0	1.8	21.1	11.2	0.001	15.0	11.2	1.8	26.3	7.1	0.002
Active Neoplasm (%)	1.8	3.6	26.3	6.7	<0.001	30.0	6.7	4.5	15.8	1.8	<0.001
Rheumatological Disease (%)	1.8	0.9	0.0	3.4	0.46	0.0	3.4	0.9	0.0	1.8	0.62
Previous Stroke/TIA (%)	1.8	0.9	5.3	1.1	0.32	5.0	1.1	0.9	5.3	1.8	0.50
Current Smoker (%)	14.0	6.3	31.6	23.6	<0.001	65.0	23.6	6.3	0.0	12.5	<0.001

Supplemental Table 3. The characteristics of SARS-CoV-2 infected stroke patients in original dataset and literature review.

Parameter	AIS-Original Data N = 323	AIS-Literature Review N= 145	P Value
Age; Mean (SD); Years	67.2 ± 15.2	63.0 ± 14.0	0.005
Sex; Men; N (%)	193 (59.8)	62 (57.4)	0.62
National Institutes of Health Stroke Scale (NIHSS); Median [IQR]	9 [4-17]	15 [9-21]	0.11
Large Vessel Occlusion; N (%)	126 (44.5)	45 (83.3)	<0.0001
Imaging Pattern of Ischemia			
Embolic/Large Vessel Athero-thromboembolism; N (%)	206 (80.8)	25 (83.4)	0.73
Lacunar; N (%)	26 (10.2)	4 (13.3)	0.60
Borderzone; N (%)	23 (9.0)	1 (3.3)	0.26
TOAST Criteria			
Large Artery Atherosclerosis; N (%)	56 (32.9)	12 (10.0)	<0.0001
Cardioembolism; N (%)	46 (27.1)	17 (14.2)	0.009
Small Artery Occlusion; N (%)	17 (10.0)	8 (6.7)	0.32
Other Determined Etiologies; N (%)	13 (7.6)	37 (30.8)	0.0001
Undetermined Etiology; N (%)	38 (22.4)	46 (38.3)	0.003
Disposition			
Discharged Home; N (%)	127 (42.8)	23 (20.2)	<0.0001
In-Hospital Mortality; N (%)	82 (27.6)	39 (34.2)	0.19
Still in Hospital, or Dispositioned to Subacute Care; N (%)	88 (29.6)	52 (45.6)	0.002
Comorbidities			
Hypertension; N (%)	202 (63.1)	64 (54.7)	0.09
Diabetes Mellitus; N (%)	111 (34.6)	43 (36.8)	0.65
Ischemic Heart Disease; N (%)	72 (24.3)	8 (6.8)	<0.001
Atrial Fibrillation; N (%)	45 (14.1)	14 (12.0)	0.53
Carotid Stenosis; N (%)	38 (12.8)	3 (2.6)	0.0006
Chronic Kidney Disease; N (%)	42 (13.1)	4 (3.4)	0.001
Cardiac Ejection Fraction <40%; N (%)	24 (8.1)	2 (1.7)	0.007
Active Neoplasm; N (%)	21 (7.1)	0 (0.0)	0.001
Rheumatological Disease; N (%)	5 (1.7)	0 (0.0)	0.11
Prior Stroke or Transient Ischemic Attack; N (%)	5 (1.7)	9 (7.6)	0.85
Smoking; N (%)	53 (16.6)	10 (8.5)	0.02
Laboratory findings			
White Blood Cell Count; Mean (SD); x109/L	9.8 ± 4.8	13.9 ± 8.7	<0.0001
Neutrophil Count; Mean (SD); x109/L	7.7 ± 4.5	10.7 ± 5.3	<0.0001
Lymphocyte Count; Mean (SD); x109/L	1.5 ± 1.5	1.2 ± 1.0	0.03
Platelet Count; Mean (SD); x109/L	314.5 ± 440.7	232 ± 110	0.03
Alanine Transaminase (ALT); Mean (SD); U/L	63.3 ± 86.3	51.0 ± 69.0	0.13
Aspartate Transaminase (AST); Mean (SD); U/L	32.1 ± 26.8	53.0 ± 43.0	<0.0001
Creatinine; Mean (SD); mg/dl	1.5 ± 1.7	1.4 ± 1.0	0.51
C-Reactive Protein (CRP); Mean (SD); mg/L	61 ± 131	99.4 ± 96.7	0.002
Lactate Dehydrogenase (LDH); Mean (SD); U/L	604.8 ± 1536.7	517.3 ± 360.3	0.50

Due to missingness, we provided the valid percentages in this table.

Supplemental 4. The characteristics of the patients from the literature review dataset divided based on clinical risk scoring models.

Parameters	Clinical Risk Scoring													
	EX-A$_2$ (All comorbidities)			EX-S$_2$ (Selected Comorbidities)			EX-A$_3$ (All comorbidities)				EX-S$_3$ (Selected Comorbidities)			
	a<br>N = 71<br>(60.7%)	b<br>N = 46<br>(39.3%)	P-value	a<br>N = 71<br>(60.7%)	b<br>N = 46<br>(39.3%)	P-value	a<br>N = 33<br>(28.2%)	b<br>N = 38<br>(32.5%)	c<br>N =46<br>(39.3%)	P-value	a<br>N = 33<br>(28.2%)	b<br>N = 38<br>(32.5%)	c<br>N =46<br>(39.3%)	P-value
**Age; Mean (SD); Years**	63±16	62±16	0.72	63±16	62±16	0.72	62±16	65±15	62±13	0.65	62±16	65±15	62±13	0.65
**Sex; Male; N (%)**	30 (53.6)	24 (68.6)	0.16	30 (53.6)	24 (68.6)	0.16	16 (59.3)	14 (48.3)	24 (68.6)	0.26	16 (59.3)	14 (48.3)	24 (68.6)	0.26
**Large Vessel Occlusion; N (%)**	22 (91.7)	18 (75.0)	0.12	22 (91.7)	18 (75.0)	0.12	8 (80.0)	14 (100.0)	18 (75.0)	0.13	8 (80.0)	14 (100.0	18 (75.0)	0.13
**Intravenous Thrombolysis; N (%)**	5 (45.5)	3 (30.0)	0.47	5 (45.5)	3 (30.0)	0.47	1 (33.3)	4 (50.0)	3 (30.0)	0.68	1 (33.3)	4 (50.0)	3 (30.0)	0.68
**Mechanical Thrombectomy; N (%)**	11 (39.3)	6 (31.6)	0.59	11 (39.3)	6 (31.6)	0.59	7 (53.8)	4 (26.7)	6 (31.6)	0.28	7 (53.8)	4 (26.7)	6 (31.6)	0.28
**National Institutes of Health Stroke Scale (NIHSS); Median [IQR]**	15 [10-19]	13 [7-23]	0.78	15 [10-19]	13 [7-23]	0.78	6 [10-19]	7 [10-16]	9 [13-20]	0.59	6 [10-19]	7 [10-16]	9 [13-20]	0.59
**TOAST Criteria**														
**Large-Artery Atherosclerosis; N (%)**	6 (10.5)	3 (7.9)		6 (10.5)	3 (7.9)		2 (6.70)	4 (14.8)	3 (7.9)		2 (6.7)	4 (14.8)	3 (7.9)	
**Cardio-embolism; N (%)**	10 (17.5)	4 (10.5)		10 (17.5)	4 (10.5)		6 (20.0)	4 (14.8)	4 (10.5)		6 (20.0)	4 (14.8)	4 (10.5)	
**Small-Vessel Occlusion; N (%)**	4 (7.00)	2 (5.30)	0.71	4 (7.00)	2 (5.30)	0.71	3 (10.0)	1 (3.7)	2 (5.3)	0.77	3 (10.0)	1 (3.7)	2 (5.3)	0.77
**Stroke of Other Determined Etiology; N (%)**	19 (33.3)	12 (31.6)		19 (33.3)	12 (31.6)		11 (36.7)	8 (29.6)	12 (31.6)		11 (36.7)	8 (29.6)	12 (31.6)	
**Stroke of Undetermined Etiology; N (%)**	18 (31.6)	17 (44.7)		18 (31.6)	17 (44.7)		8 (26.7)	10 (37.0)	17 (44.7)		8 (26.7)	10 (37.0)	17 (44.7)	
**Imaging Patterns**														
**Embolic-Appearing; N (%)**	7 (77.8)	12 (85.7)		7 (77.8)	12 (85.7)		2 (66.7)	5 (83.3)	12 (85.7)		2 (66.7)	5 (83.3)	12 (85.7)	
**Lacune; N (%)**	1 (11.1)	2 (14.3)	0.44	1 (11.1)	2 (14.3)	0.44	0 (0.00)	1 (16.7)	2 (14.3)	0.12	0 (0.00)	1 (16.7)	2 (14.3)	0.12
**Borderzone; N (%)**	1 (11.1)	0 (0.00)		1 (11.1)	0 (0.00)		1 (33.3)	0 (0.0)	0 (0.0)		1 (33.3)	0 (0.0)	0 (0.0)	
**Interval Between Infection Onset to Stroke; Median [IQR]; Days**	6 [2-16]	7 [2-14]	0.40	6 [2-16]	7 [2-14]	0.40	6 [1-6]	9 [5-20]	6 [2-14]	0.03	6 [1-6]	9 [5-20]	6 [2-14]	0.03
**Mechanical Ventilation; N (%)**	10 (25.0)	4 (20.0)	0.42	10 (25.0)	4 (20.0)	0.42	7 (33.3)	3 (15.8)	4 (20.0)	0.32	7 (33.3)	3 (15.8)	4 (20.0)	0.32
**Disposition**														
**Discharged Home; N (%)**	14 (24.6)	5 (13.5)		14 (24.6)	5 (13.5)		6 (22.2)	8 (26.7)	5 (13.5)		6 (22.2)	8 (26.7)	5 (13.5)	
**In Hospital Mortality; N (%)**	17 (29.8)	14 (37.8)	0.40	17 (29.8)	14 (37.8)	0.40	8 (29.6)	9 (30.0)	14 (37.8)	0.73	8 (29.6)	9 (30.0)	14 (37.8)	0.73
**Still in Hospital/Subacute Care; N (%)**	26 (45.6)	18 (48.6)		26 (45.6)	18 (48.6)		13 (48.1)	13 (43.3)	18 (48.6)		13 (48.1)	13 (43.[illegible]	18 (48.6)	
**Comorbidities**														
**Hypertension; N (%)**	20 (28.2)	44 (95.7)	<0.001	20 (28.2)	44 (95.7)	<0.001	0 (0.0)	20 (52.6)	44 (95.7)	<0.001	0 (0.0)	20 (52.6)	44 (95.7)	<0.001
**Diabetes Mellitus; N (%)**	10 (14.1)	33 (71.7)	<0.001	10 (14.1)	33 (71.7)	<0.001	0 (0.0)	10 (26.3)	33 (71.7)	<0.001	0 (0.0)	10 (26.3)	33 (71.7)	<0.001
**Ischemic Heart Disease; N (%)**	1 (1.40)	7 (15.2)	0.004	1 (1.40)	7 (15.2)	0.004	0 (0.0)	1 (2.6)	7 (15.2)	0.01	0 (0.0)	1 (2.6)	7 (15.2)	0.01
**Atrial Fibrillation; N (%)**	2 (2.80)	12 (26.1)	<0.001	2 (2.80)	12 (26.1)	<0.001	0 (0.0)	2 (5.3)	12 (26.1)	0.001	0 (0.0)	2 (5.3)	12 (26.1)	0.001
**Carotid Stenosis; N (%)**	0 (0.00)	3 (6.50)	0.03	0 (0.00)	3 (6.5)	0.03	0 (0.0)	0 (0.0)	3 (6.50)	0.09	0 (0.0)	0 (0.0)	3 (6.5)	0.09
**Chronic Kidney Disease; N (%)**	0 (0.00)	4 (8.70)	0.01	0 (0.00)	4 (8.7)	0.01	0 (0.0)	0 (0.0)	4 (8.70)	0.04	0 (0.0)	0 (0.0)	4 (8.7)	0.04
**Cardiac Ejection Fraction <40%; N (%)**	0 (0.00)	1 (2.20)	0.21	0 (0.00)	1 (2.2)	0.21	0 (0.0)	0 (0.0)	1 (2.20)	0.46	0 (0.0)	0 (0.0)	1 (2.2)	0.46
**Active Neoplasm; N (%)**	0 (0.00)	0 (0.00)	-	0 (0.00)	0 (0.00)	-	0 (0.0)	0 (0.0)	0 (0.00)	1.00	0 (0.0)	0 (0.0)	0 (0.0)	1.00
**Rheumatological Disease; N (%)**	0 (0.00)	0 (0.00)	-	0 (0.00)	0 (0.00)	-	0 (0.0)	0 (0.0)	0 (0.00)	1.00	0 (0.0)	0 (0.0)	0 (0.0)	1.00
**Prior Stroke or Transient Ischemic Attack; N (%)**	0 (0.00)	9 (19.6)	<0.001	0 (0.00)	9 (19.6)	<0.001	0 (0.0)	0 (0.0)	9 (19.6)	0.001	0 (0.0)	0 (0.0)	9 (19.6)	0.001
**Smoking; N (%)**	5 (7.00)	5 (10.9)	0.47	5 (7.00)	5 (10.9)	0.47	0 (0.0)	5 (13.2)	5 (10.9)	0.11	0 (0.0)	5 (13.2)	5 (10.9)	0.11

EX-A$_2$: clinical risk-scoring (expert opinion) model including all comorbidities; a, 0-1 comorbidity; b, >1 comorbidity; EX-S$_2$: clinical risk-scoring model including selected comorbidities; a, 0-1 comorbidity; b, >1 comorbidity; EX-A$_3$: clinical risk scoring model including all comorbidities; a, 0 comorbidity; b, 1-2 comorbidities, c, >2 comorbidities; EX-S$_3$: clinical risk scoring model including selected comorbidities; a, 0 comorbidity; b, 1-2 comorbidities, c, >2 comorbidities. Due to missingness, we provided the valid percentages in this table.

SUPPLEMENTAL 5. THE CHARACTERISTICS OF THE PATIENTS FROM THE LITERATURE REVIEW DATASET DIVIDED BASED ON UNSUPERVISED MACHINE LEARNING MODELS.

Parameter	Machine Learning Algorithms													
	**ML-K_2 (K-Mean)**			**ML-S_2 (Spectral)**			**ML-K_3 (K-Mean)**				**ML-S_3 (Spectral)**			
	**a N = 50 (42.8%)**	**b N = 67 (57.2%)**	**P-value**	**a N = 10 (8.5%)**	**b N =107 (91.5%)**	**P-value**	**a N = 50 (42.7%)**	**b N = 48 (41.0%)**	**c N = 19 (16.2%)**	**P-value**	**a N = 10 (8.5%)**	**b N = 77 (65.8%)**	**c N = 30 (25.6%)**	**P-value**
**Age; Mean (SD); Years**	62±15	63±15	0.86	53±6	63±15	0.34	57±16	64±15	62±15	0.42	53±6	64±14	61±18	0.45
**Sex; Male; N (%)**	21 (46.7)	24 (53.3)	0.37	10 (100.0)	46 (56.8)	<0.001	24 (63.2)	20 (51.3)	4 (66.7)	0.52	2 (100.0)	26 (51.0)	20 (66.7)	0.18
**Large Vessel Occlusion; N (%)**	15 (88.2)	25 (82.1)	0.58	0 (0.0)	40 (84.4)	<0.001	15 (88.2)	19 (82.6)	4 (21.1)	0.85	0 (0.0)	27 (84.4)	11 (84.6)	0.98
**Intravenous Thrombolysis; N (%)**	2 (40.0)	6 (40.0)	0.99	0 (0.0)	8 (40.0)	<0.001	2 (40.0)	4 (36.4)	2 (10.5)	0.89	0 (0.0)	7 (53.8)	1 (14.3)	0.09
**Mechanical Thrombectomy; N (%)**	8 (42.1)	9 (34.6)	0.61	0 (0.0)	17 (37.8)	<0.001	8 (42.1)	9 (40.9)	0 (0.00)	0.26	0 (0.0)	10 (31.3)	7 (53.8)	0.16
**National Institutes of Health Stroke Scale (NIHSS); Median [IQR]**	14 [10-16]	18 [7-21]	0.51	-	16 [8-21]		9 [10-16]	9 [7-21]	11 [6-21]	0.22	-	10 [6-24]	-	-
**TOAST Criteria**														
**Large-Artery Atherosclerosis; N (%)**	3 (6.8)	6 (11.8)		0 (0.0)	9 (9.7)		3 (6.80)	4 (10.3)	1 (9.1)		1 (25.0)	5 (7.90)	3 (10.7)	
**Cardio-embolism; N (%)**	7 (15.9)	7 (13.7)		0 (0.0)	14 (15.1)		7 (15.9)	5 (12.8)	2 (18.2)		0 (0.0)	10 (15.9)	4 (14.3)	
**Small-Vessel Occlusion; N (%)**	4 (9.1)	2 (3.9)	0.81	0 (0.0)	6 (6.4)	0.37	4 (9.10)	2 (5.1)	0 (0.0)	0.94	0 (0.0)	4 (6.3)	2 (7.1)	0.43
**Stroke of Other Determined Etiology; N (%)**	15 (34.1)	16 (31.4)		2 (100.0)	29 (31.2)		15 (34.1)	13 (33.3)	3 (27.3)		3 (75.0)	23 (36.5)	5 (17.9)	
**Stroke of Undetermined Etiology; N (%)**	15 (34.1)	20 (39.2)		0 (0.0)	35 (37.6)		15 (34.1)	15 (38.5)	5 (45.5)		0 (0.0)	21 (33.3)	14 (50.0)	
**Imaging Patterns**														
**Embolic-Appearing; N (%)**	3 (60.0)	16 (88.9)		-	19 (82.6)		3 (60.0)	11 (84.6)	5 (100.0)		3 (100.0)	3 (60.0)	13 (86.7)	
**Lacune; N (%)**	1 (20.0)	2 (11.1)	0.18	-	3 (13.0)	<0.001	1 (20.0)	2 (15.4)	0 (0.0)	0.44	0 (0.0)	1 (20.0)	2 (13.3)	0.18
**Borderzone; N (%)**	1 (20.0)	0 (0.0)		-	1 (4.4)		1 (20.0)	0 (0.00)	0 (0.0)		0 (0.0)	1 (20.0)	0 (0.00)	
**Interval Between Infection Onset to Stroke; Median [IQR]; Days**	5 [1-7]	9 [2-15]	0.01	-	6 [2-15]		7 [1-7]	7 [5-15]	8 [1-10]	0.003	-	8 [2-15]	6 [2-14]	0.033
**Mechanical Ventilation; N (%)**	10 (20.0)	4 (14.3)	0.08	1 (100.0)	13 (22.8)	0.21	10 (33.3)	3 (12.0)	1 (33.3)	0.21	1 (100.0)	7 (16.7)	6 (40.0)	0.16
**Disposition**														
**Discharged Home; N (%)**	9 (21.9)	10 (14.9)		0 (0.0)	19(20.4)		9 (22.0)	6 (15.0)	4 (28.6)		3 (42.8)	11 (17.7)	5 (20.0)	
**In Hospital Mortality; N (%)**	11 (26.8)	20 (37.7)	0.54	0 (0.0)	31 (33.3)	0.60	11 (26.8)	14 (35.0)	7 (50.0)	0.84	3 (42.8)	19 (30.6)	9 (36.0)	0.84
**Still in Hospital/Subacute Care; N (%)**	21 (42.0)	23 (34.3)		1 (100.0)	43 (46.3)		21 (51.2)	20 (50.0)	3 (21.4)		1 (14.2)	32 (51.6)	11 (44.0)	
**Comorbidities**														
**Hypertension; N (%)**	0 (0.0)	64 (95.5)	<0.001	0 (0.0)	64 (59.8)	<0.001	0 (0.0)	47 (97.9)	17 (89.5)	<0.001	0 (0.0)	45 (58.4)	19 (63.3)	0.16
**Diabetes Mellitus; N (%)**	10 (20.0)	33 (49.2)	0.002	0 (0.0)	43 (36.4)	<0.001	10 (20.0)	21 (43.8)	12 (63.2)	<0.001	0 (0.0)	32 (42.6)	11 (36.7)	0.57
**Ischemic Heart Disease; N (%)**	2 (4.0)	6 (9.0)	0.34	0 (0.0)	8 (7.5)	0.71	2 (4.0)	3 (6.30)	3 (15.8)	0.22	0 (0.0)	5 (6.5)	3 (10.0)	0.62
**Atrial Fibrillation; N (%)**	3 (6.0)	11 (18.6)	0.05	0 (0.0)	14 (13.1)	<0.001	3 (6.0)	0 (0.0)	11 (57.9)	<0.001	0 (0.0)	11 (14.3)	3 (10.0)	0.72
**Carotid Stenosis; N (%)**	0 (0.0)	4 (6.0)	0.11	0 (0.0)	3 (2.8)	0.81	0 (0.0)	3 (6.3)	0 (0.0)	0.14	0 (0.0)	2 (2.6)	1 (3.30)	0.95
**Chronic Kidney Disease; N (%)**	0 (0.0)	4 (6.0)	0.11	0 (0.0)	4 (3.7)	0.89	0 (0.0)	1 (2.1)	3 (15.8)	0.53	0 (0.0)	3 (3.9)	1 (3.30)	0.27
**Cardiac Ejection Fraction <40%; N (%)**	0 (0.0)	1 (1.5)	0.35	0 (0.0)	1 (0.9)	0.89	0 (0.0)	0 (0.0)	1 (5.3)	0.01	0 (0.0)	0 (0.0)	1 (3.30)	0.27
**Active Neoplasm; N (%)**	0 (0.0)	0 (0.0)	-	0 (0.0)	0 (0.0)	-	0 (0.0)	0 (0.0)	0 (0.0)	1.00	0 (0.0)	0 (0.0)	0 (0.00)	1.00
**Rheumatological Disease; N (%)**	0 (0.0)	0 (0.0)	-	0 (0.0)	0 (0.0)	-	0 (0.0)	0 (0.0)	0 (0.0)	1.00	0 (0.0)	0 (0.0)	0 (0.00)	1.00
**Prior Stroke or Transient Ischemic Attack; N (%)**	0 (0.0)	9 (13.4)	0.01	0 (0.0)	9 (8.4)	<0.001	0 (0.0)	6 (12.5)	3 (15.8)	0.04	0 (0.0)	5 (6.5)	4 (13.3)	0.19
**Smoking; N (%)**	4 (8.0)	6 (8.9)	0.54	0 (0.0)	10 (9.3)	<0.001	4 (8.0)	3 (6.3)	3 (15.8)	0.62	0 (0.0)	6 (7.8)	4 (13.3)	0.19

ML-K_2: machine learning model using K-mean, dividing the patients into 2 subgroups; ML-S_2: machine learning model using spectral, dividing the patients into 2 subgroups; ML-K_3: machine learning model using K-mean, dividing the patients into 3 subgroups; ML-S_3: machine learning model using spectral, dividing the patients into 3 subgroups. Please note a, b, and c in this table are not based on the number of comorbidities and just indicated a distinct subgroup detected by unsupervised algorithms. Due to missingness, we provided the valid percentages in this table.

SUPPLEMENTAL FIGURE 1. CONTINGENCY MATRICES

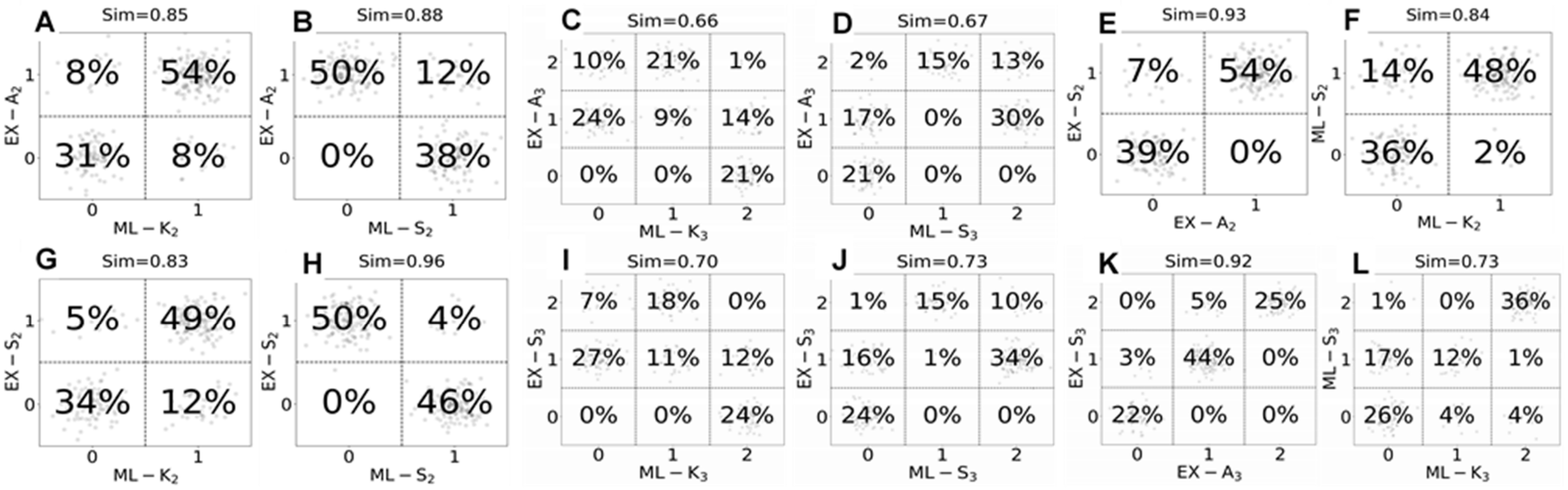

Supplemental Figure 1. The similarity of methods under different clustering models when grouping the patients with acute ischemic stroke into 2 and 3 subgroups. In models based on expert opinion (EX), EX-A included all 11 collected comorbidities and EX-S included 8 selected comorbidities. Patients were then clustered into two subgroups (EX-A_2 and EX-S_2), with “0” referring to patients with zero or one comorbidities and “1” referring to patients with >1 comorbidity, or three subgroups (EX-A_3 and EX-S_3), with “0” referring to patients without any known comorbidity, “1” referring to patients with one or two comorbidities, and “2” referring to patients with >2 comorbidities. In machine learning algorithms (ML) ML-K used hierarchal clustering and K-means, while ML-S used spectral clustering. Within each clustering, method patients were grouped into two (ML-K_2 and ML-S_2) and three (ML-K_3 and ML-S_3) clusters. Panel **A** shows the similarity between the 2 subgroups in EX-A_2 and ML-K_2. Panel **B** shows the similarity between the 2 subgroups in EX-A_2 and ML-S_2. Panel **C** shows the similarity between the 3 subgroups in EX-A_3 and ML-K_3. Panel **D** shows the similarity between the 3 subgroups in EX-A_3 and ML-S_3. Panel **E** shows the similarity between the 2 subgroups in EX-S_2 and EX-A_2. Panel **F** shows the similarity between the 2 subgroups in ML-S_2 and ML-K_2. Panel **G** shows the similarity between the 2 subgroups in EX-S_2 and ML-K_2. Panel **H** shows the similarity between the 2 subgroups in EX-S_2 and ML-S_2. Panel **I** shows the similarity between the 3 subgroups in EX-S_3 and ML-K_3. Panel **J** shows the similarity between the 3 subgroups in EX-S_3 and ML-S_3. Panel **K** shows the similarity between the 3 subgroups in EX-S_3 and EX-A_3. Panel **L** shows the similarity between the 3 subgroups in ML-S_3 and ML-K_3.

The COVID-19 Stroke Study Group

Shima Shahjouei, MD, MPH,[1] Georgios Tsivgoulis, MD, PhD, MSc,[2] Ghasem Farahmand, MD,[3,4] Eric Koza, MD Candidate,[5] Ashkan Mowla, MD,[1,6] Alireza Vafaei Sadr, PhD,[7] Arash Kia, MD,[8] Alaleh Vaghefi Far, MD,[4] Stefania Mondello, MD, PhD, MPH,[9] Achille Cernigliaro, PhD, MPH,[10] Annemarei Ranta, MD, PhD, FRACP,[11] Martin Punter, PhD, MBChB,[11] Faezeh Khodadadi, Pharm.D,[12] Soheil Naderi, MD,[13] Mirna Sabra, PhD,[14] Mahtab Ramezani, MD,[15] Ali Amini Harandi, MD,[15] Oluwaseyi Olulana, MS,[5] Durgesh Chaudhary, MBBS,[1] Aicha Lyoubi, MD,[16] Bruce Campbell, MD,[17,18] Juan F. Arenillas, MD,[19] Daniel Bock, MD,[20] Joan Montaner, MD,[21] Saeideh Aghayari Sheikh Neshin, MD,[22] Diana Aguiar de Sousa, MD, PhD,[23] Matthew S. Tenser, MD,[6] Ana Aires, MD,[24,25] Merccedes De Lera Alfonso, MD,[19] Orkhan Alizada,[26] MD, Elsa Azevedo, MD,[24,25], Nitin Goyal, MD,[27] Zabihollah Babaeepour, MD,[28] Gelareh Banihashemi, MD,[29] Leo H. Bonati, MD,[30] Carlo Cereda, MD,[31] Jason J. Chang, MD,[32] Miljenko Crnjakovic, MD,[33] GianMarco De Marchis, MD,[30] Christoph Griessenauer, MD,[1,34] Michelle Anyaehie, MBS,[1] Massimo Del Sette, MD,[35] Seyed Amir Ebrahimzadeh, MD, MPH,[36] Mehdi Farhoudi, MD,[37] Elyar Sadeghi-Hokmabadi,[37] Ilaria Gandoglia, MD,[35] Bruno Gonçalves, MD,[38] Mehmet Murat Hancı, MD,[26] Aristeidis H. Katsanos, MD,[2,39] Christos Krogias, MD,[40] Ronen Leker, MD,[41] Lev Lotman, MD,[42] Jeffrey Mai, MD,[43] Shailesh Male, MD,[44] Konark Malhotra, MD,[45] Branko Malojcic, MD, PhD,[46] Teresa Mesquita, MD,[47] Asadollah Mirghasemi, MD,[48] Hany Mohamed Aref, MD,[49] Zeinab Mohseni Afshar, MD,[50] Jusun Moon, MD,[51] Mika Niemelä, MD, PhD,[52] Behnam Rezai Jahromi, MD, [52] Lawrence Nolan, MD,[41] Abhi Pandhi, MD,[27] Jong-Ho Park, MD,[53] João Pedro Marto, MD,[46] Francisco Purroy, MD, PhD[54] Sakineh Ranji-Burachaloo, MD,[3] Nuno Reis Carreira, MD,[55] Manuel Requena, MD,[56] Marta Rubiera, MD,[56] Seyed Aidin Sajedi, MD,[57] João Sargento-Freitas, MD, [58] Vijay Sharma, MD,[59] Thorsten Steiner, MD,[60,61] Kristi Tempro, MD,[41] Guillaume Turc,[38] MD, PhD, FESO, Yassaman Ahmadzadeh, MD,[62] Mostafa Almasi-Dooghaee, MD,[63,64] Farhad Assarzadegan, MD,[14] Arefeh Babazadeh, MD, MPH,[65] Humain Baharvahdat, MD,[66] Fabricio Cardoso, MD, MPH,[67] Apoorva Dev, PhD,[12] Mohammad Ghorbani, MD,[68] Ava Hamidi, MD,[69] Zeynab Sadat Hasheminejad, MD,[70] Sahar Hojjat-Anasri Komachali, MD,[71] Fariborz Khorvash, MD,[72] Firas Kobeissy, PhD,[73] Hamidreza Mirkarimi, MD,[74] Elahe Mohammadi-Vosough, MD,[74] Debdipto Misra, MS,[75] Ali Reza Noorian, MD,[76] Peyman Nowrouzi-Sohrabi, PhD,[77] Sepideh Paybast, MD,[78] Leila Poorsaadat, MD,[79] Mehrdad Roozbeh, MD,[80] Behnam Sabayan, MD, PhD,[81] Saeideh Salehizadeh,[82] Alia Saberi, MD,[22] Mercedeh Sepehrnia, MD,[70] Fahimeh Vahabizad, MD,[29] Thomas Yasuda, MD,[67] Mojdeh Ghabaee, MD[3,4], Nasrin Rahimian, MD, MPH,[83] Mohammad Hossein Harirchian, MD,[3] Afshin Borhani-Haghighi, MD,[84] Mahmoud Reza Azarpazhooh, MD,[85] Rohan Arora, MD,[86] Saeed Ansari, MD,[27] Venkatesh Avula, MS,[87] Jiang Li, MD,[88] Vida Abedi, MD,[87,88] Ramin Zand, MD, MPH.[1]

[1]Neurology Department, Neuroscience Institute, Geisinger Health System, Danville, Pennsylvania, USA;

[2]Second Department of Neurology, National and Kapodistrian University of Athens, School of Medicine, "Attikon" University Hospital, Athens, Greece;

[3]Iranian Center of Neurological Research, Neuroscience Institute, Tehran University of Medical Sciences, Tehran, Iran;

[4]Neurology Department, Tehran University of Medical Sciences, Tehran, Iran;

[5]Geisinger Commonwealth School of Medicine, Scranton, Pennsylvania, USA;

[6]Division of Stroke and Endovascular Neurosurgery, Department of Neurological Surgery, Keck School of Medicine, University of Southern California, California, USA;

[7]Department de Physique Theorique and Center for Astroparticle Physics, University Geneva, Switzerland;

[8]Icahn school of medicine at Mount Sinai, Department of Population Health Science and Policy, Institute for Healthcare Delivery Science, New York, USA;

[9]Department of Biomedical and Dental Sciences and Morphofunctional Imaging, University of Messina, Messina, Italy;

[10]Regional Health Authority of Sicily, Palermo, Italy;

[11]Department of Neurology, Wellington Hospital, Wellington, New Zealand;

[12]PES University, Bangaluru, Karnataka, India;

[13]Department of Neurosurgery, Tehran University of Medical Sciences, Tehran, Iran;

[14]Neurosciences Research Center (NRC), Lebanese University/ Medical School, Beirut, Lebanon;

[15]Neurology Department, Shahid Beheshti University of Medical Sciences, Tehran, Iran;

[16]Neurology Department, Delafontaine Hospital, Saint-Denis, France;

[17]Department of Neurology, Royal Melbourne Hospital, Parkville, Australia;

[18]University of Melbourne, Parkville, Australia;

[19]Department of Neurology, University of Valladolid, Valladolid, Spain;

[20]Department of Cardiology, Klinikum Frankfurt Höchst, Frankfurt, Germany;

[21]Department of Neurology, Hospital Universitario Virgen Macarena, Sevilla, Spain;

[22]Neurology Department, Poursina Hospital, Rasht, Guilan, Iran;

[23]Department of Neurology, Hospital de Santa Maria, University of Lisbon, Lisbon, Portugal;

[24]Department of Neurology, Centro Hospitalar Universitário de São João, Porto, Portugal;

[25]Faculty of Medicine, University of Porto, Porto, Portugal;

[26]Neurosurgery Department, Istanbul University-Cerrahpasa, Cerrahpasa Medical Faculty, Istanbul, Turkey;

[27]Department of Neurology, University of Tennessee, Tennessee, USA;

[28]Neurology Ward, Valiasr Hospital, Borujen, Iran;

[29]Neurology Department, Sina Hospital, Tehran University of Medical Sciences, Tehran, Iran;

[30]Department of Neurology and Stroke Unit, University Hospital Basel, Switzerland;

[31]Stroke Center, Neurocenter of Southern Switzerland, Lugano, Switzerland;

[32]Department of Critical Care Medicine, MedStar Washington Hospital Center, Washington, DC, USA;

[33]Intensive Care Unit, Department of Neurology, Clinical Hospital Dubrava, Zagreb, Croatia;

[34]Research Institute of Neurointervention, Paracelsus Medical University, Salzburg, Austria;

[35]Neurology Unit, Galliera Hospital, Genova, Italy;

[36]Department of Radiology, Yasrebi Hospital, Kashan, Iran;

[37]Neurosciences Research Center (NSRC), Tabriz University of Medical Sciences, Tabriz, Iran;

[38]Department of Neurology, GHU Paris Psychiatrie et Neurosciences, Université de Paris, INSERM U1266, Paris, France;

[39]Division of Neurology, McMaster University/ Population Health Research Institute, Hamilton, ON, Canada;

[40]Department of Neurology, St. Josef-Hospital, Ruhr University Bochum, Bochum, Germany;

[41]Department of Neurology, Hadassah-Hebrew University Medical Center, Jerusalem, Israel;

[42]Department of Neurology, Albany Medical Center, Albany, NY;

[43]Department of Neurosurgery, Georgetown University and MedStar Washington Hospital Center, Washington, DC, USA;

[44]Neurology Department, Vidant Medical Center, Greenville, North Carolina, USA;

[45]Department of Neurology, Allegheny Health Network (AHN), Pittsburgh, Pennsylvania, USA;

[46]TIA Clinic, Department of Neurology, University Hospital Centre Zagreb, Zagreb School of Medicine, University of Zagreb, Zagreb, Croatia;

[47]Department of Neurology, Hospital de Egas Moniz, Centro Hospitalar Lisboa Ocidental, Lisbon, Portugal;

[48]Department of Anesthesiology, University of Ottawa, Canada;

[49]Department of Neurology, Ain Shams University, Cairo, Egypt;

[50]Infection Disease Research Center, Kermanshah University of Medical Sciences, Kermanshah, Iran;

[51]Department of Neurology, National Medical Center, Seoul, South Korea;

[52]Department of Neurosurgery, Helsinki University Hospital, Helsinki, Finland;

[53]Department of Neurology, Myongji Hospital, Hanyang University College of Medicine, South Korea;

[54]Department of Neurology, Hospital Arnau de Vilanova, Lleida, Spain;

[55]Department of Neurology, Hospital Arnau de Vilanova, Institut de Recerca Biomèdica de Lleida (IRBLLeida), Universitat de Lleida UdL Lleida, Spain;

[56]Stroke Unit, Department of Neurology, Hospital Vall d'Hebron, Department de Medicina, Universitat Autònoma de Barcelona, Barcelona, Spain;

[57]Neuroscience Research Center, Department of Neurology, Golestan University of Medical Sciences, Golestan, Iran;

[58]Department of Neurology, Centro Hospitalar e Universitário de Coimbra, Coimbra, Portugal;

[59]Division of Neurology, University Medicine Cluster, National University Health System, Singapore, Singapore;

[60]Department of Neurology, Klinikum Frankfurt Höchst, Frankfurt, Germany;

[61]Department of Neurology Heidelberg University Hospital, Germany;

[62] Hospital for Special Surgery, New York, USA;

[63]Divisions of Vascular and Endovascular Neurosurgery and Neurology, Firoozgar Hospital, Iran University of Medical Sciences, Tehran, Iran;

[64]Divisions of Vascular and Endovascular Neurosurgery and Neurology, Rasoul-Akram Hospital, Iran University of Medical Sciences, Tehran, Iran;

[65]Infectious Diseases and Tropical Medicine Research Center, Health Research Institute, Babol University of Medical Sciences, Babol, Iran;

[66]Neurosurgical Department, Ghaem Hospital, Mashhad University of Medical Sciences, Mashhad, Iran;

[67]Neurology Department, Centro Médico de Campinas, São Paulo, Brazil;

[68]Division of Vascular and Endovascular Neurosurgery, Firoozgar Hospital, Iran University of Medical Sciences, Tehran, Iran;

[69]Neurology Ward, Gheshm Hospital, Gheshm, Iran;

[70]Department of Neurology, Imam Hosein Hospital, Shahid Beheshti Medical University, Tehran, Iran;

[71]Department of Neurology, Pirooz hospital, Gilan University of Medical Sciences, Lahijan, Iran;

[72]Professor Of Neurology, Isfahan University of Neurology, Isfahan, Iran;

[73]Program of Neurotrauma, Neuroproteomics and Biomarker Research (NNBR), University of Florida, Florida, USA;

[74]Neurology Ward, Modarres Hospital, Kashmar, Iran;

[75]Steele Institute of Health & Innovation, Geisinger Health System, Pennsylvania, USA;

[76]Department of Neurology, Southern California Permanente Medical Group, Irvine, California, USA;

[77]Student Research Committee, Shiraz University of Medical Sciences, Shiraz, Iran;

[78]Department of Neurology, Bou Ali hospital, Qazvin University of Medical Sciences, Qazvin, Iran;

[79]Department of Neurology, Arak University of Medical Sciences, Arak, Iran;

[80]Brain Mapping Research Center, Shahid Beheshti University of Medical Sciences, Tehran, Iran;

[81]Department of Neurology, Massachusetts General Hospital, Harvard Medical School, Boston, MA, USA;

[82]Salahadin Ayubi Hospital, Baneh, Iran;

[83]Department of Neurology, Yasrebi Hospital, Kashan, Iran;

[84]Clinical Neurology Research Center, Shiraz University of Medical Sciences, Shiraz, Iran;

[85]Department of Clinical Neurological Sciences, Western University, Canada;

[86]Department of Neurology, Long Island Jewish Forest Hills, Queens, New York, USA;

[87]Department of Molecular and Functional Genomics, Geisinger Health System, Danville, Pennsylvania, USA;

[88]Biocomplexity Institute, Virginia Tech, Blacksburg, Virginia, USA.
